# 2-(4-Hydroxy­phenyl­sulfon­yl)phenol

**DOI:** 10.1107/S160053680900258X

**Published:** 2009-01-28

**Authors:** Kazuyuki Sato, Hideki Shima, Jin Mizuguchi

**Affiliations:** a Department of Applied Physics, Graduate School of Engineering, Yokohama National University, 79-5 Tokiwadai, Hodogaya-ku, 240-8501 Yokohama, Japan

## Abstract

The title compound, C_12_H_10_O_4_S, is a phenolic color developer used for leuco colorants. The two benzene rings with substituent hydr­oxy groups are nearly perpendicular to each other [dihedral angle = 91.5 (1)°]. There are inter­molecular O—H⋯O hydrogen bonds between an OH group of one mol­ecule and a sulfonyl O atom of a neighboring mol­ecule. One mol­ecule is hydrogen bonded to four symmetry-related mol­ecules, forming a two-dimensional hydrogen-bond network.

## Related literature

For general background literature on leuco dyes, see: Muthyala (1997 ▶). For the structure of 4,4′-sulfonyl­diphenol, see: Glidewell & Ferguson (1996 ▶); Davies *et al.* (1997 ▶).
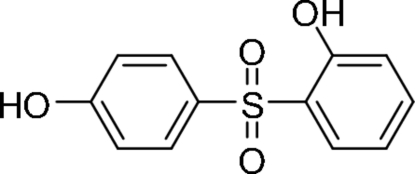


## Experimental

### 

#### Crystal data

C_12_H_10_O_4_S
*M* *_r_* = 250.27
Monoclinic, 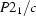

*a* = 10.9525 (2) Å
*b* = 14.4404 (3) Å
*c* = 7.0361 (1) Å
β = 93.8147 (10)°
*V* = 1110.35 (3) Å^3^
*Z* = 4
Cu- *K*α radiation
μ = 2.62 mm^−1^
*T* = 93.1 K
0.39 × 0.35 × 0.29 mm

#### Data collection

Rigaku R-AXIS RAPID diffractometer
Absorption correction: multi-scan (*ABSCOR*; Higashi, 1995 ▶) *T*_min_ = 0.408, *T*_max_ = 0.468
9436 measured reflections
1998 independent reflections
1830 reflections with *F* ^2^ > 2σ(*F* ^2^)
*R*_int_ = 0.154

#### Refinement

*R*[*F* ^2^ > 2σ(*F* ^2^)] = 0.058
*wR*(*F* ^2^) = 0.162
*S* = 1.10
1998 reflections
163 parameters
H atoms treated by a mixture of independent and constrained refinement
Δρ_max_ = 0.55 e Å^−3^
Δρ_min_ = −0.66 e Å^−3^

### 

Data collection: *PROCESS-AUTO* (Rigaku, 1998 ▶); cell refinement: *PROCESS-AUTO*; data reduction: *CrystalStructure* (Rigaku/MSC, 2006 ▶); program(s) used to solve structure: *SIR2004* (Burla *et al.*, 2005 ▶); program(s) used to refine structure: *SHELXL97* (Sheldrick, 2008 ▶); molecular graphics: *ORTEPIII* (Burnett & Johnson, 1996 ▶); software used to prepare material for publication: *CrystalStructure*.

## Supplementary Material

Crystal structure: contains datablocks global, I. DOI: 10.1107/S160053680900258X/bh2215sup1.cif

Structure factors: contains datablocks I. DOI: 10.1107/S160053680900258X/bh2215Isup2.hkl

Additional supplementary materials:  crystallographic information; 3D view; checkCIF report

## Figures and Tables

**Table 1:** Hydrogen-bond geometry (Å, °)

*D*—H⋯*A*	*D*—H	H⋯*A*	*D*⋯*A*	*D*—H⋯*A*
O3—H3O⋯O2^i^	0.87 (3)	1.90 (3)	2.753 (2)	168 (3)
O4—H4O⋯O1^ii^	0.88 (4)	1.85 (4)	2.733 (2)	173 (3)

Symmetry codes: (i) 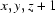; (ii) 
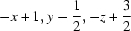
.

**Fig. 1.**
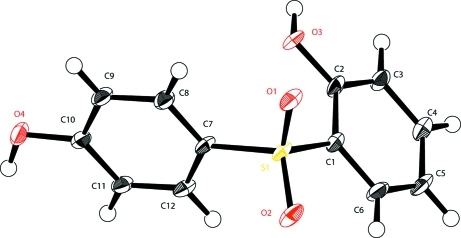
A view of the molecular structure of (I), showing 50% probability displacement ellipsoids for non-H atoms.

**Fig. 2.**
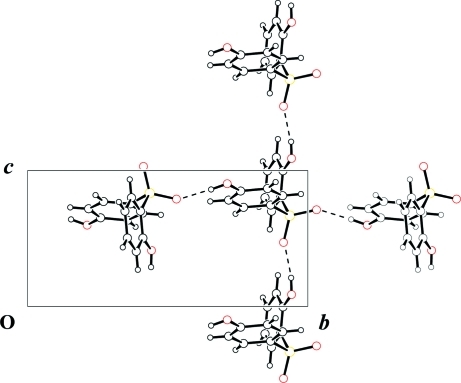
Projection of the structure of (I) onto the b and c plane, showing O—H···O intermolecular hydrogen bonds in dotted lines. One molecule is hydrogen-bonded to four different molecules along the b and c axes.

## supplementary crystallographic information

### Comment

The colorless leuco dye is known to exhibit a brilliant color when the lactone-ring is opened by the formation of intermolecular hydrogen bonds between dye and developer, and is used in practice in thermal or rewritable papers (Muthyala, 1997). The title compound has found use as a developer for fluoran leuco dyes which give a black color. Since the color is generated by a solid state reaction by heating a mixture of dye and developer particles, the mutual geometrical relation of dye and developer molecules plays an important role in color strength in the solid state. For this reason, structure analysis of 2-[4-hydroxyphenyl]sulfonyl]-phenol, (I), has been carried out in the present investigation.

Figure 1 shows the *ORTEPIII* plot (Burnett & Johnson, 1996) of (I). The two benzene rings with 2-hydroxy or 4-hydroxy group are nearly perpendicular to each other [dihedral angle: 91.5 (1)°]. Figure 2 is the projection of the crystal structure onto the (*b,c*) plane. There are O—H···O intermolecular hydrogen bonds between the OH of one molecule and the sulfonyl O atom of the neighboring one along the *b* and *c* axes. One molecule is hydrogen-bonded to four different molecules, forming a two-dimensional hydrogen-bond network. A similar two-dimensional network is found in 4,4'-sulfonyldiphenol (Glidewell & Ferguson, 1996; Davies *et al.*, 1997).

### Experimental

Compound (I) was obtained from Mitsubisihi Paper Mills., Ltd., and was recrystallized from an ethanol solution. After 48 h., a number of colorless crystals were obtained in the form of blocks.

### Refinement

The H atoms of the hydroxy groups (H3O and H4O) were found in density maps and refined isotropically. All other H atoms were positioned geometrically and included in the riding-model approximation, with C—H distances of 0.95 Å, and *U*_iso_(H) = 1.2*U*_eq_(C).

### Crystal data

**Table:**

C_12_H_10_O_4_S	*F*(000) = 520.00
*M**_r_* = 250.27	*D*_x_ = 1.497 Mg m^−^^3^
Monoclinic, *P*2_1_/*c*	Cu *K*α radiation, λ = 1.54187 Å
Hall symbol: -P 2ybc	Cell parameters from 9882 reflections
*a* = 10.9525 (2) Å	θ = 3.1–68.2°
*b* = 14.4404 (3) Å	µ = 2.62 mm^−^^1^
*c* = 7.0361 (1) Å	*T* = 93 K
β = 93.8147 (10)°	Block, colorless
*V* = 1110.35 (3) Å^3^	0.39 × 0.35 × 0.29 mm
*Z* = 4	

### Data collection

**Table:**

Rigaku R-AXIS RAPID diffractometer	1830 reflections with *F*^2^ > 2σ(*F*^2^)
Detector resolution: 10.00 pixels mm^-1^	*R*_int_ = 0.154
ω scans	θ_max_ = 68.2°
Absorption correction: multi-scan (*ABSCOR*; Higashi, 1995)	*h* = −12→12
*T*_min_ = 0.408, *T*_max_ = 0.468	*k* = −17→17
9436 measured reflections	*l* = −8→8
1998 independent reflections	

### Refinement

**Table:**

Refinement on *F*^2^	H atoms treated by a mixture of independent and constrained refinement
*R*[*F*^2^ > 2σ(*F*^2^)] = 0.058	*w* = 1/[σ^2^(*F*_o_^2^) + (0.078*P*)^2^ + 1.2611*P*] where *P* = (*F*_o_^2^ + 2*F*_c_^2^)/3
*wR*(*F*^2^) = 0.162	(Δ/σ)_max_ < 0.001
*S* = 1.10	Δρ_max_ = 0.55 e Å^−^^3^
1998 reflections	Δρ_min_ = −0.66 e Å^−^^3^
163 parameters	Extinction correction: *SHELXL97* (Sheldrick, 2008)
0 restraints	Extinction coefficient: 0.0078 (10)
0 constraints	

### Fractional atomic coordinates and isotropic or equivalent isotropic displacement parameters (Å^2^)

**Table:**

	*x*	*y*	*z*	*U*_iso_*/*U*_eq_	
S1	0.27104 (6)	0.93583 (5)	0.66743 (9)	0.0192 (2)	
O1	0.30244 (18)	1.03113 (13)	0.7107 (3)	0.0240 (5)	
O2	0.23614 (19)	0.91341 (15)	0.4710 (2)	0.0272 (5)	
O3	0.25813 (18)	0.94202 (14)	1.0879 (2)	0.0217 (4)	
O4	0.70235 (18)	0.71229 (14)	0.9019 (3)	0.0253 (5)	
C1	0.1463 (2)	0.90270 (18)	0.7994 (3)	0.0184 (5)	
C2	0.1531 (2)	0.91128 (18)	0.9972 (4)	0.0201 (6)	
C3	0.0508 (2)	0.8876 (2)	1.0952 (4)	0.0222 (6)	
C4	−0.0549 (2)	0.8560 (2)	0.9967 (4)	0.0243 (6)	
C5	−0.0613 (2)	0.8473 (2)	0.7997 (4)	0.0265 (6)	
C6	0.0399 (2)	0.8704 (2)	0.7008 (4)	0.0243 (6)	
C7	0.3959 (2)	0.86727 (19)	0.7456 (3)	0.0192 (5)	
C8	0.5037 (2)	0.90989 (19)	0.8143 (4)	0.0209 (6)	
C9	0.6055 (2)	0.85644 (19)	0.8666 (4)	0.0218 (6)	
C10	0.5990 (2)	0.76019 (19)	0.8485 (3)	0.0199 (6)	
C11	0.4904 (2)	0.71764 (18)	0.7819 (3)	0.0204 (6)	
C12	0.3887 (2)	0.77072 (19)	0.7286 (3)	0.0213 (6)	
H3O	0.257 (3)	0.940 (2)	1.212 (5)	0.023 (8)*	
H4O	0.695 (4)	0.653 (3)	0.862 (6)	0.058 (12)*	
H3	0.0535	0.8933	1.2300	0.025*	
H4	−0.1237	0.8395	1.0653	0.027*	
H5	−0.1341	0.8255	0.7318	0.028*	
H6	0.0369	0.8644	0.5664	0.027*	
H8	0.5071	0.9755	0.8264	0.022*	
H9	0.6792	0.8851	0.9143	0.025*	
H11	0.4865	0.6518	0.7720	0.023*	
H12	0.3143	0.7422	0.6813	0.024*	

### Atomic displacement parameters (Å^2^)

**Table:**

	*U*^11^	*U*^22^	*U*^33^	*U*^12^	*U*^13^	*U*^23^
S1	0.0162 (4)	0.0227 (4)	0.0174 (4)	−0.0020 (2)	−0.0079 (2)	0.0010 (2)
O1	0.0209 (10)	0.0219 (10)	0.0280 (10)	−0.0042 (7)	−0.0088 (8)	0.0019 (8)
O2	0.0222 (11)	0.0383 (11)	0.0199 (10)	−0.0033 (8)	−0.0087 (8)	0.0052 (9)
O3	0.0172 (10)	0.0281 (10)	0.0185 (10)	−0.0040 (7)	−0.0089 (8)	0.0000 (8)
O4	0.0167 (10)	0.0220 (10)	0.0359 (11)	0.0023 (7)	−0.0089 (8)	−0.0010 (9)
C1	0.0140 (12)	0.0198 (12)	0.0206 (13)	−0.0011 (9)	−0.0050 (10)	0.0013 (11)
C2	0.0167 (13)	0.0180 (12)	0.0240 (14)	0.0030 (10)	−0.0100 (10)	0.0040 (11)
C3	0.0193 (13)	0.0265 (13)	0.0197 (13)	0.0024 (10)	−0.0071 (10)	0.0018 (11)
C4	0.0169 (13)	0.0278 (14)	0.0273 (15)	−0.0001 (10)	−0.0056 (10)	0.0028 (12)
C5	0.0186 (14)	0.0295 (15)	0.0295 (15)	−0.0054 (11)	−0.0123 (11)	−0.0008 (12)
C6	0.0211 (14)	0.0299 (14)	0.0204 (13)	−0.0023 (11)	−0.0091 (10)	−0.0026 (12)
C7	0.0172 (13)	0.0230 (13)	0.0160 (12)	−0.0017 (10)	−0.0081 (10)	0.0002 (11)
C8	0.0190 (14)	0.0189 (12)	0.0240 (14)	−0.0028 (10)	−0.0039 (10)	−0.0024 (11)
C9	0.0188 (14)	0.0204 (13)	0.0251 (14)	−0.0042 (10)	−0.0064 (10)	−0.0032 (11)
C10	0.0191 (13)	0.0213 (13)	0.0186 (12)	−0.0001 (10)	−0.0049 (10)	−0.0026 (11)
C11	0.0216 (14)	0.0181 (12)	0.0206 (13)	−0.0031 (10)	−0.0050 (10)	−0.0032 (10)
C12	0.0206 (13)	0.0222 (13)	0.0201 (13)	−0.0057 (10)	−0.0063 (10)	−0.0024 (11)

### Geometric parameters (Å, °)

**Table:**

S1—O1	1.446 (2)	C4—H4	0.952
S1—O2	1.446 (2)	C5—C6	1.388 (4)
S1—C1	1.768 (2)	C5—H5	0.955
S1—C7	1.747 (2)	C6—H6	0.948
O3—C2	1.353 (3)	C7—C8	1.389 (3)
O3—H3O	0.87 (3)	C7—C12	1.401 (3)
O4—C10	1.358 (3)	C8—C9	1.386 (3)
O4—H4O	0.90 (4)	C8—H8	0.951
C1—C2	1.395 (3)	C9—C10	1.397 (3)
C1—C6	1.396 (3)	C9—H9	0.948
C2—C3	1.397 (4)	C10—C11	1.392 (3)
C3—C4	1.386 (3)	C11—C12	1.383 (3)
C3—H3	0.950	C11—H11	0.953
C4—C5	1.389 (4)	C12—H12	0.953
			
O1···O4^i^	2.733 (2)	O3···H8^ii^	2.862
O1···H4O^i^	1.84 (4)	O3···H9^ii^	2.590
O1···H9^ii^	2.898	O3···H12^v^	2.799
O1···H11^i^	2.891	O4···O1^vi^	2.733 (2)
O2···O3^iii^	2.753 (2)	O4···H4^vii^	2.835
O2···H3O^iii^	1.89 (3)	O4···H5^vii^	2.757
O2···H3^iii^	2.552	O4···H5^viii^	2.888
O3···O2^iv^	2.753 (2)	C4···H9^ix^	2.962
			
O1—S1—O2	117.29 (12)	C6—C5—H5	119.7
O1—S1—C1	109.16 (12)	C1—C6—C5	119.9 (2)
O1—S1—C7	107.63 (12)	C1—C6—H6	119.9
O2—S1—C1	106.08 (12)	C5—C6—H6	120.2
O2—S1—C7	108.99 (12)	S1—C7—C8	119.2 (2)
C1—S1—C7	107.30 (12)	S1—C7—C12	119.9 (2)
C2—O3—H3O	113 (2)	C8—C7—C12	120.8 (2)
C10—O4—H4O	110 (2)	C7—C8—C9	119.7 (2)
S1—C1—C2	120.6 (2)	C7—C8—H8	120.0
S1—C1—C6	118.6 (2)	C9—C8—H8	120.3
C2—C1—C6	120.9 (2)	C8—C9—C10	119.6 (2)
O3—C2—C1	119.1 (2)	C8—C9—H9	120.1
O3—C2—C3	122.2 (2)	C10—C9—H9	120.2
C1—C2—C3	118.7 (2)	O4—C10—C9	116.4 (2)
C2—C3—C4	120.3 (2)	O4—C10—C11	123.1 (2)
C2—C3—H3	119.9	C9—C10—C11	120.5 (2)
C4—C3—H3	119.8	C10—C11—C12	120.1 (2)
C3—C4—C5	120.9 (2)	C10—C11—H11	119.8
C3—C4—H4	119.4	C12—C11—H11	120.1
C5—C4—H4	119.6	C7—C12—C11	119.3 (2)
C4—C5—C6	119.3 (2)	C7—C12—H12	120.1
C4—C5—H5	120.9	C11—C12—H12	120.6
			
O1—S1—C1—C2	−55.8 (2)	C6—C1—C2—C3	−0.5 (3)
O1—S1—C1—C6	122.4 (2)	O3—C2—C3—C4	−179.7 (2)
O1—S1—C7—C8	−6.2 (2)	C1—C2—C3—C4	0.1 (3)
O1—S1—C7—C12	177.4 (2)	C2—C3—C4—C5	0.0 (3)
O2—S1—C1—C2	177.0 (2)	C3—C4—C5—C6	0.1 (3)
O2—S1—C1—C6	−4.9 (2)	C4—C5—C6—C1	−0.4 (4)
O2—S1—C7—C8	122.0 (2)	S1—C7—C8—C9	−176.4 (2)
O2—S1—C7—C12	−54.5 (2)	S1—C7—C12—C11	176.7 (2)
C1—S1—C7—C8	−123.5 (2)	C8—C7—C12—C11	0.3 (4)
C1—S1—C7—C12	60.0 (2)	C12—C7—C8—C9	0.1 (3)
C7—S1—C1—C2	60.6 (2)	C7—C8—C9—C10	0.4 (4)
C7—S1—C1—C6	−121.3 (2)	C8—C9—C10—O4	179.8 (2)
S1—C1—C2—O3	−2.5 (3)	C8—C9—C10—C11	−1.3 (4)
S1—C1—C2—C3	177.6 (2)	O4—C10—C11—C12	−179.6 (2)
S1—C1—C6—C5	−177.5 (2)	C9—C10—C11—C12	1.6 (4)
C2—C1—C6—C5	0.6 (4)	C10—C11—C12—C7	−1.1 (4)
C6—C1—C2—O3	179.4 (2)		

Symmetry codes: (i) −*x*+1, *y*+1/2, −*z*+3/2; (ii) −*x*+1, −*y*+2, −*z*+2; (iii) *x*, *y*, *z*−1; (iv) *x*, *y*, *z*+1; (v) *x*, −*y*+3/2, *z*+1/2; (vi) −*x*+1, *y*−1/2, −*z*+3/2; (vii) *x*+1, *y*, *z*; (viii) *x*+1, −*y*+3/2, *z*+1/2; (ix) *x*−1, *y*, *z*.

### Hydrogen-bond geometry (Å, °)

**Table:**

*D*—H···*A*	*D*—H	H···*A*	*D*···*A*	*D*—H···*A*
O3—H3O···O2^iv^	0.87 (3)	1.90 (3)	2.753 (2)	168 (3)
O4—H4O···O1^vi^	0.88 (4)	1.85 (4)	2.733 (2)	173 (3)

Symmetry codes: (iv) *x*, *y*, *z*+1; (vi) −*x*+1, *y*−1/2, −*z*+3/2.

## References

[bb1] Burla, M. C., Caliandro, R., Camalli, M., Carrozzini, B., Cascarano, G. L., De Caro, L., Giacovazzo, C., Polidori, G. & Spagna, R. (2005). *J. Appl. Cryst.* **38**, 381–388.

[bb2] Burnett, M. N. & Johnson, C. K. (1996). *ORTEPIII* Report ORNL-6895. Oak Ridge National Laboratory, Tennessee, USA.

[bb3] Davies, C., Langler, R. F., Sharma, C. V. K. & Zaworotko, M. J. (1997). *Chem. Commun.* pp. 567–568.

[bb4] Glidewell, C. & Ferguson, G. (1996). *Acta Cryst.* C**52**, 2528–2530.

[bb5] Higashi, T. (1995). *ABSCOR* Rigaku Corporation, Tokyo, Japan.

[bb6] Muthyala, R. (1997). In *Chemistry and Applications of Leuco Dyes* New York, London: Plenum Press.

[bb7] Rigaku (1998). *PROCESS-AUTO* Rigaku Corporation, Tokyo, Japan.

[bb8] Rigaku/MSC (2006). *CrystalStructure* Rigaku/MSC, The Woodlands, Texas, USA.

[bb9] Sheldrick, G. M. (2008). *Acta Cryst.* A**64**, 112–122. 10.1107/S0108767307043930 18156677

